# Repurposing antiviral phytochemicals from the leaf extracts of *Spondias mombin* (Linn) towards the identification of potential SARSCOV-2 inhibitors

**DOI:** 10.1038/s41598-022-14558-3

**Published:** 2022-06-28

**Authors:** Akwasi Boadu, Clement Agoni, Rajshekhar Karpoormath, Mahmoud Soliman, Manimbulu Nlooto

**Affiliations:** 1 Discipline of Pharmaceutical Sciences, School of Health Sciences, University of KwaZulu-Natal, Durban, 4000 South Africa; 2 Department of Pharmacy, School of Health Care Sciences, University of Limpopo, Private Bag X1106, Polokwane, Sovenga, 0727 South Africa; 3 Molecular Bio-Computation and Drug Design Laboratory, School of Health Sciences, Discipline of Pharmaceutical Sciences, University of KwaZulu-Natal, KwaZulu-Natal, South Africa; 4 Synthetic and Medicinal Chemistry Research Group (SMCRG), Department of Pharmaceutical Chemistry, Discipline of Pharmaceutical Sciences, School of Health Sciences, University of KwaZulu-Natal, Durban, 4000 South Africa

**Keywords:** Computational biology and bioinformatics, Drug discovery, Chemistry

## Abstract

Severe Acute Respiratory Syndrome Coronavirus 2 (SARS-CoV-2), a pneumonia-like disease with a pattern of acute respiratory symptoms, currently remains a significant public health concern causing tremendous human suffering. Although several approved vaccines exist, vaccine hesitancy, limited vaccine availability, high rate of viral mutation, and the absence of approved drugs account for the persistence of SARS-CoV-2 infections. The investigation of possibly repurposing of phytochemical compounds as therapeutic alternatives has gained momentum due to their reported affordability and minimal toxicity. This study investigated anti-viral phytochemical compounds from ethanolic leaf extracts of *Spondias mombin* L as potential inhibitor candidates against SARS-CoV-2. We identified Geraniin and 2-O-Caffeoyl-(+)-allohydroxycitric acid as potential SARS-CoV-2 inhibitor candidates targeting the SARS-CoV-2 RNA-dependent polymerase receptor-binding domain (RBD) of SARS-CoV-2 viral S-protein and the 3C-like main protease (3CL^pro^). Geraniin exhibited binding free energy (ΔGbind) of − 25.87 kcal/mol and − 21.74 kcal/mol towards SARS-CoV-2 RNA-dependent polymerase and receptor-binding domain (RBD) of SARS-CoV-2 viral S-protein respectively, whereas 2-O-Caffeoyl-(+)-allohydroxycitric acid exhibited a ΔGbind of − 32 kcal/mol towards 3CL^pro^. Molecular Dynamics simulations indicated a possible interference to the functioning of SARS-CoV-2 targets by the two identified inhibitors. However, further in vitro and in vivo evaluation of these potential SARS-CoV-2 therapeutic inhibitor candidates is needed.

## Introduction

Reported in Wuhan, China, in early December 2019, Severe Acute Respiratory Syndrome Coronavirus 2 (SARS-CoV-2) infection has spread worldwide and was eventually declared a pandemic by the World Health Organisation (WHO) on March 11, 2020^1^. As of 7th January 2022, coronavirus disease (COVID-19) had a 2% mortality rate and over 360 million infected people worldwide^2–8^.

SARS-CoV-2, belonging to the genus Betacoronavirus^9,10^ is known to infect individuals through contact with body fluids and secretions such as respiratory droplets^2,11–25^. The SARS-CoV-2 disease is characterised by acute respiratory problems, fever, cough, sore throat, loss of taste, smell and myalgia, kidney failure, pneumonia, and death in severe forms of the disease^2,4^.

Several vaccines have been developed and approved for use by the WHO as the number of infections continues to increase^26–30^. Notable vaccines^31^ being administered in many parts of the world include; mRNA-1273 (Moderna)^32^, BNT162b2 (Pfizer/BioNTech)^33,34^, ChAdOx1 nCoV-19 (Astrazenac/Oxford)^5,35^, (rAd26)/rAd5 (Sputnik V)^5,36,37^, and Janssen Ad26.COV2.S (Johnson and Johnson)^38^. Targeted therapeutics such Chloroquine, Hydroxychloroquine, Remdesivir (GS‐5734), Favipiravir, Ivermectin and Lopinavir/Ritonavir has also been extensively investigated and repurposed for the treatment of SARS-CoV-2 based on their previously reported potential as antiviral therapeutics^39–46^. The Food and Drug Administration subsequently approved Remdesivir, Ronapreve^30^ to treat COVID-19 in hospitalized adults and pediatric patients^47,48^. Nonetheless, the search for novel treatment options continues, whereby several therapeutic targets of SARS-CoV-2^49–55^ have been thoroughly explored.

Several phytochemicals from natural products are being investigated as potential therapeutic in the treatment of SARS-CoV-2^56–59^, due to their affordability, effectiveness, safety, cultural preferences, and ample accessibility^60–63^. Notable of such recent reports is the study by van Breemen et al. (2022)^64^ where cannabinoids were shown to block cellular entry of SARS-CoV-2 and the emerging variants. This report amongst many others has identified many potential SARS-CoV-2 inhibitors from natural sources^63,65–67^.

Indigenous to tropical African and South American countries^68^, *Spondias mombin Linn* (*S. mombin*) has been ethnomedicinally used to treat viral infections^57–60,69–81^, respiratory tract infections^56^ and inflammatory disorders^73,82–85^. Of the numerous phytochemicals isolated from the leaf extracts of *S. mombin*, some reportedly possess anti-viral activities (Table 1), although, the therapeutic potential of these phytochemical compounds isolated from *S. mombin* remains unexplored.

**Table 1:** Selected phytochemicals from alcoholic extracts of the leaves of *S. mombin* with reported anti-viral properties.

No.	Name of compound	Pharmacological action	References
1	Geraniin	Anti-viral properties against Dengue virus type-2 (DENV-2), Zika (ZIKV) virus, hepatitis B virus, and herpes simplex virus type 1, Coxsackie B virus	^86–94^
2	3,5-di-O-galloyl-4-O-digalloylquinic acid	Human immunodeficiency virus (HIV) reverse transcriptase (RT)	^95^
3	3-O-digalloyl-4,5-di-O-galloylquinic acid	Anti-viral activity against Human immunodeficiency virus (HIV) reverse transcriptase (RT)	^95^
4	1,3,4,5-tetra-O-galloylquinic acid	Anti-viral activity against Human immunodeficiency virus (HIV) reverse transcriptase (RT)	^95^
5	2-O-Caffeoyl-(+)-allohydroxycitric acid	Antiviral activities against Coxsackie B virus	^70^
6	6-(8'Z,11'Z,14'Z-heptadecatrienyl)-salicylic acid	Anti-malarial properties against *Mycobacterium fortuitum* and chloroquine-sensitive strains of *Plasmodium falciparum*	^96^
7	6-(10'Z-heptadecenyl)-salicylic acid	Anti-plasmodial properties against *Mycobacterium fortuitum* and chloroquine-sensitive strains of *Plasmodium falciparum*	^96^

This current study seeks to employ computational techniques to investigate the potential of these anti-viral extracts of *S. mombin* as inhibitory agents against the SARS-CoV-2 RNA-dependent polymerase^97–101^, 3C-like main protease (3CL^pro^)^52,102–106^, and the receptor-binding domain of the viral S-protein of the SARS-CoV-2^55^. We also employed in silico methods to thoroughly assess drug-likeness of the compounds and complement our findings with molecular dynamics (MD) simulations to unravel conformational perturbations associated with the potential inhibitory activity of the identified bioactive compounds. Although in silico approaches as employed in this study are inconclusive, they could accelerate the discovery of viable anti-SARS-CoV-2 therapeutics and at a low cost.

## Computational methodology

### System preparation

The X-ray crystal structures of SARS-CoV-2 RNA-dependent polymerase (PDB:7BTF)^107^, SARS-CoV-2 3C-like main protease (3CL^pro^) (PDB:6LU7)^108^, and the receptor-binding domain of viral S-protein (PDB:6M17)^109^ were retrieved from the Protein Data Bank^110^ and prepared for molecular docking using and UCSF Chimera^111^.

### Retrieval and preparation of investigated phytochemicals

The anti-viral phytochemicals investigated in this study (Table 1) were drawn on Marvin Sketch^112^ to generate their 2D structures. Subsequently, energy minimization and optimization of the 2D structures of phytochemicals were performed using Avogadro 1.2.0 software^113^ and the UFF force field with the steepest descent algorithm^109^. Afterward, the 3D of the structures of each compound was generated and saved as mol2 files for further investigations.

### Binding pocket identification and molecular docking of modeled structures into SARS-CoV-2 therapeutic targets

The bound co-crystallized inhibitors were used to map out the respective binding pockets for the investigated SARS-CoV-2 therapeutic targets. Mapping-out of the binding pocket was performed using the grid box function in AutoDock Vina^114^, whereby respective coordinate which denotes the respective binding pocket of the therapeutic targets were generated. The grid box coordinates for the inhibitor binding site of SARS-CoV-2 RNA-dependent RNA polymerase were calculated as; X = 124.119, Y = 124.064, Z = 133.126 (center) and X = 22.935, Y = 29.805, Z = 19.22 (size). Grid box coordinates for SARS-CoV-2 3CL^pro^ were calculated as X = − 12.333, Y = 13.837, Z = 64.250 (centre), and X = 15.396, Y = 14.656, Z = 15.085 (size). The grid box coordinates for the inhibitor binding pocket of the receptor-binding domain (RBD) of viral S-protein were also calculated as X = 28.346, Y = 22.296, Z = 25.538 (center), and X = 178.034, Y = 122.853, Z = 244.886 (size). Subsequently, molecular docking of the generated bioactive compounds was then carried out using AutoDock Vina with exhaustiveness of 8. Results of the molecular docking were viewed using the ViewDock function incorporated in UCSF Chimera. The docking results were validated by superimposing generated docked complexes with the retrieved co-crystallized structures of the target SARS-CoV-2 therapeutic targets.

### In silico exploration of drug-likeness of hits

We predicted the physicochemical and pharmacokinetic properties of the studied phytochemicals such as absorption, distribution, metabolism, excretion, and toxicity (ADMET) using SwissADME^115^. The drug-likeness of the phytochemicals were also determined by predicting the adherence of each compound to Lipinski's rules of five^116^, which is widely employed in assessing the drug-likeness chemical compounds^117^. Although these properties could be evaluated using experimental methods, they are usually time-consuming and expensive^118–120^.

### Molecular dynamics (MD) simulations

To reveal the conformational and structural changes that accompany the binding of the investigated phytochemical compounds, we performed an atomic-scale MD simulation using the AMBER 18 GPU with an integrated Particle Mesh Ewald Molecular Dynamics (PMEMD) module^121,122^. These structural changes could inform the possible inhibitory mechanism of the identified compounds. The ANTECHAMBER module was used to parameterize the inhibitors, atomic partial charges (AM1BCC) were added^123^. The FF14SB AMBER force field was also used to parameterize the retrieved structures^124^. Protonation of histidine residues was then performed using the pdb4amber script at a constant pH (cpH) to ensure compatibility of the prepared SARS-CoV-2 therapeutic target models with the LEAP module. Subsequently, the LEAP module was employed to solvate and neutralize the entire prepared system. The counter ions, Na^+^ or Cl^−^ were used to neutralize as systems whereas TIP3P orthorhombic box size of 12 Å of water molecules was added to solvate each system^125^. Topology and coordinate files of the bioactive compounds, SARS-CoV-2 therapeutic targets, and the resultant complexes were then generated and saved. The prepared bound complexes and the unbound therapeutic targets were then subjected to an initial 2000 minimization steps with a restraint potential of 500 kcal/mol; afterward, a 1000 steps steepest descent minimization with no restraint was performed on the entire system. Each system was gradually heated from 0 to 300 K for 50 ps. After heating, a 500 ps equilibration was performed at a constant pressure of 1 bar using Berendsen barostat^126^. The SHAKE algorithm was employed to constrict all atomic hydrogen bonds, after which a 200 ns MD simulation was performed using a 1 fs time step^127^. Coordinates for generated MD trajectories were saved at 1 ps intervals. These generated trajectories were further analysed using the PTRAJ and CPPTRAJ modules of AMBER18^128^. Graphical plots for analysis of the generated trajectories created with the Microcal Origin analytical software^129^.

### Binding free energy calculations

Binding free energies were calculated using the Molecular Mechanics/Poisson-Boltzmann Surface Area (MM/PBSA) techniques implemented in AMBER18^130,131^, a technique that determines structural stability, predicts binding affinities and hotspots. This technique has been widely applied in protein–ligand interactions with proven reliability over the years. The binding free energies (ΔGbind) was determined by the equations:

1 $$\Delta {\text{Gbind}} = {\text{Gcomplex}}{-}\left( {{\text{Greceptor}} + {\text{Gligand}}} \right)$$

2 $$\Delta {\text{G}}_{{{\text{bind}}}} = \Delta {\text{H}} - {\text{T}}\Delta {\text{S}} = \Delta {\text{E}}_{{{\text{MM}}}} + \Delta {\text{G}}_{{{\text{sol}}}} - {\text{T}}\Delta {\text{S}}$$

where

3 $$\Delta {\text{E}}_{{{\text{MM}} }} = \Delta {\text{E}}_{{{\text{int}}}} + \Delta {\text{E}}_{{{\text{vdw}}}} + \Delta {\text{E}}_{{{\text{elec}}}}$$

4 $${\text{G}}_{{{\text{sol}}}} = {\text{G}}_{{{\text{PB}}}} + {\text{G}}_{{{\text{SA}}}}$$

5 $${\text{G}}_{{{\text{non}}\_{\text{polar}}}} =\upgamma {\text{SASA}} + \beta$$

where ΔE_MM_, ΔG_sol_, and ΔS are the changes in the gas phase molecular mechanics (MM) energy, solvation free energy, and conformational entropy upon ligand binding. ΔE_int_ refers to the energies of bond, angle, and torsion, whereas ΔE_vdw_ denotes van der Waals energies. The non-bonded electrostatic energy components are also denoted by ΔE_elec_. The solvation free energy, G_sol_, on the other hand, is a summation of the electrostatic solvation energy ΔG_PB_ (polar contribution) and the nonpolar contribution ΔG_SA_ between the solute and the continuum solvent. G_SA_ is calculated from the solvent assessable surface area (SASA), obtained by means of a 1.4 Å water probe radius, whereas the polar contribution is calculated using Poisson–Boltzmann (PB). γ and β are empirical constants of 0.00542 kcal/(mol Å^2^) and 0.92 kcal/mol, respectively. Frames employed in the binding free energy calculations included only frames generated after systems had stabilized.

## Results and discussion

### Molecular docking of the anti-viral phytochemical from ethanolic leaf extracts of S. mombin with SARS-CoV-2 RNA dependent RNA polymerase, SARS-CoV-2 3CL^pro^ and RBD of viral S-protein

The molecular docking technique was used to explore the inhibitory potential of the phytochemical compounds from ethanolic leaf extract of *S. mombin* against SARS-CoV-2 therapeutic targets, molecular docking was performed. The docking scores, which gave insights into the possible binding affinity of the compounds against the studied targets, were calculated as presented in Table 2.

**Table 2:** Docking scores of selected anti-viral phytochemical compounds and reference drugs against SARS-CoV-2 RdRp, 3CL^pro^ and RBD of viral S-protein.

The reported anti-viral phytochemical compounds from *S. mombin*	SARS-CoV-2 RNA-dependent RNA polymerase (kcal/mol)	Receptor binding domain (RBD) of viral S-protein (kcal/mol)	SARS-CoV-2 3C-like main protease (kcal/mol)
Geraniin	− 10.4	− 7.3	31.2
6-(8′Z, 11′Z, 14′Z-heptadecatrienyl)-salicylic acid	− 5.1	− 4.9	− 5.2
2-O-Caffeoyl-(+)-allohydroxycitric acid	− 6.8	− 5.6	− 5.6
3,5-di-O-galloyl-4-O-digalloylquinic acid	− 9.0	− 7.2	− 0.5
3,4-di-O-galloyl-5-O-digalloylquinic acid	− 8.3	− 6.0	1.1
3-O-digalloyl-4,5-di-O-galloylquinic acid	9.1	− 6.4	− 3.9
Remdesivir	− 8.2	–	–
Ritonavir	–	–	− 5.5

Key: compound was not docked against target (–).

Docking scores allow for the determination of the most favourable binding orientation of a compound within a given binding pocket. A favourable binding orientation of a ligand within a given pocket influences the nature of binding interaction and hence influences overall binding affinity^132^. Reports from other authors indicated that the lower the docking score, the more favourable the corresponding binding orientation^132^. As shown in Table 2, molecular docking of all the studied compounds at the active site of SARS-CoV-2 revealed that Geraniin exhibited the most favourable binding orientation at the inhibitor binding sites of both SARS-CoV-2 RdRp and the RBD of viral S-protein with the highest docking score of − 10.4 kcal/mol and − 7.3 kcal/mol respectively. Also, at the inhibitor binding site of 3CL^pro^, 2-O-Caffeoyl-(+)-allohydroxycitric acid exhibited the highest docking score of − 5.6 kcal/mol against binding to 3CL^pro^.

### Exploring the binding mechanisms of identified hit phytochemicals against SARS-CoV-2 therapeutic targets

The binding mechanisms of inhibitors to biological targets are usually characterized by interactions that exist between the inhibitor and amino acids that constitute the binding site of the biological targets. These interactions consequentially influence the conformational dynamics of the biological target as well as the stability and binding affinity of the inhibitors. Therefore, inhibitor-residue interactions are very crucial in the overall therapeutic potential of inhibitors. Using the Discovery Studio^133^, we visualized and explored the residue interaction profile of Geraniin and 2-O-Caffeoyl-(+)-allohydroxycitric acid upon binding to SARS-CoV-2 3CL^pro^, SARS-CoV-2 RdRp, and the RBD of viral S-protein. Molecular insights from the binding interactions as explored herein could shed more light on the binding potential binding mechanisms of the investigated phytochemicals.

#### SARS-CoV-2 RNA 3 C-like main protease-2-O-Caffeoyl-(+)-allohydroxycitric acid-binding mechanism

**Figure 1:**
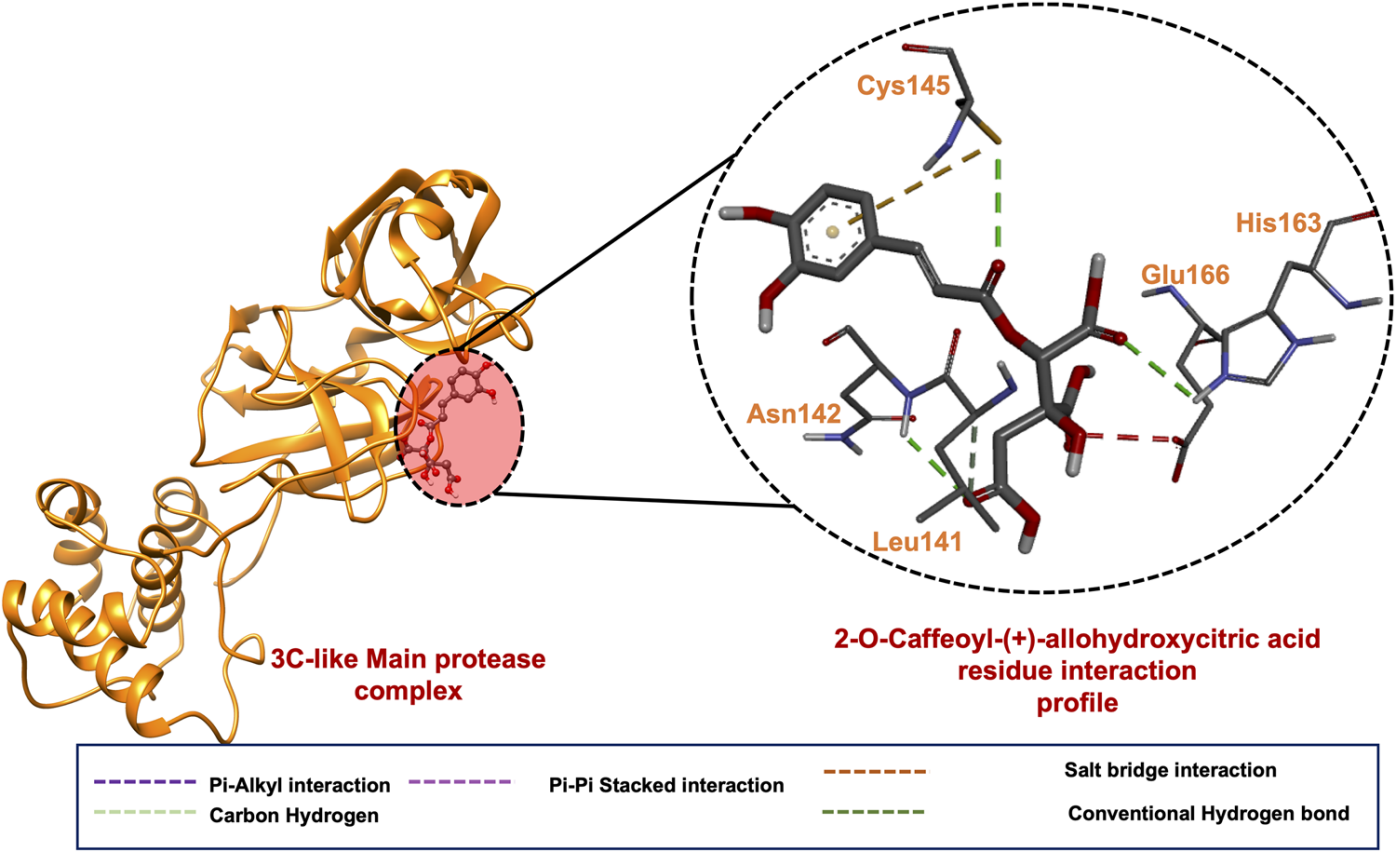
(**A**) A 3D complex of 3CL^pro^-2-O-Caffeoyl-(+)-allohydroxycitric acid. (**B**) A 3D ligand interaction plot of the RdRp-2-O-Caffeoyl-(+)-allohydroxycitric acid complex.

After exhibiting the most favourable docking scores towards SARS-CoV-2 3CL^pro^ amongst all the investigated compounds, as shown in Table 2, we analysed the possible binding mechanisms of 2-O-Caffeoyl-(+)-allohydroxycitric acid its interaction profile with binding site residues. As shown in Fig. 1, an analysis of the binding interactions of 2-O-Caffeoyl-(+)-allohydroxycitric acid towards 3CL^pro^ indicated the formation of strong intermolecular interactions with crucial binding sites residues. Notably, strong conventional hydrogen bond interactions formed with Csy145, Asn142 and His163. Cys145 is shown to engage in an additional pi-cation interaction with the bound inhibitor emphasizing its cruciality to the binding of 2-O-Caffeoyl-(+)-allohydroxycitric acid. A study by Hall et al*.* (2020)^134^ reported that His163 is essential to the inhibition of 3CL^pro^ since the mutation of its homologous residue His162 in SARS-CoV-2 protease inactivates 3CL^pro^^134^. As such, the conventional hydrogen bond interaction engaged between 2-O-Caffeoyl-(+)-allohydroxycitric acid and His163 further highlights this residue's cruciality and also predicts a possible -inhibitory potential of 2-O-Caffeoyl-(+)-allohydroxycitric acid against 3CL^pro^. 2-O-Caffeoyl-(+)-allohydroxycitric acid was also observed to engage in conventional hydrogen bond interaction with Cys145, one of the catalytic dyad (Cys145 and His41)^135^ of 3CL^pro^. The therapeutic modulation of the catalytic dyad has been reported to impact its catalytic activity and overall conformational fold of 3CL^pro^ due to the role of the catalytic dyad in facilitating the cleavage of SARS-CoV-2 polyproteins^136^. Therefore, the observed high-affinity hydrogen bond interaction between 2-O-Caffeoyl-(+)-allohydroxycitric acid and Cys145 suggested its possible inhibitory modulation of the catalytic dyad thereby warranting its further investigations as a potential inhibitor of 3CL^pro^.

#### SARS-CoV-2 RNA dependent RNA polymerase-Geraniin complex binding mechanism

**Figure 2:**
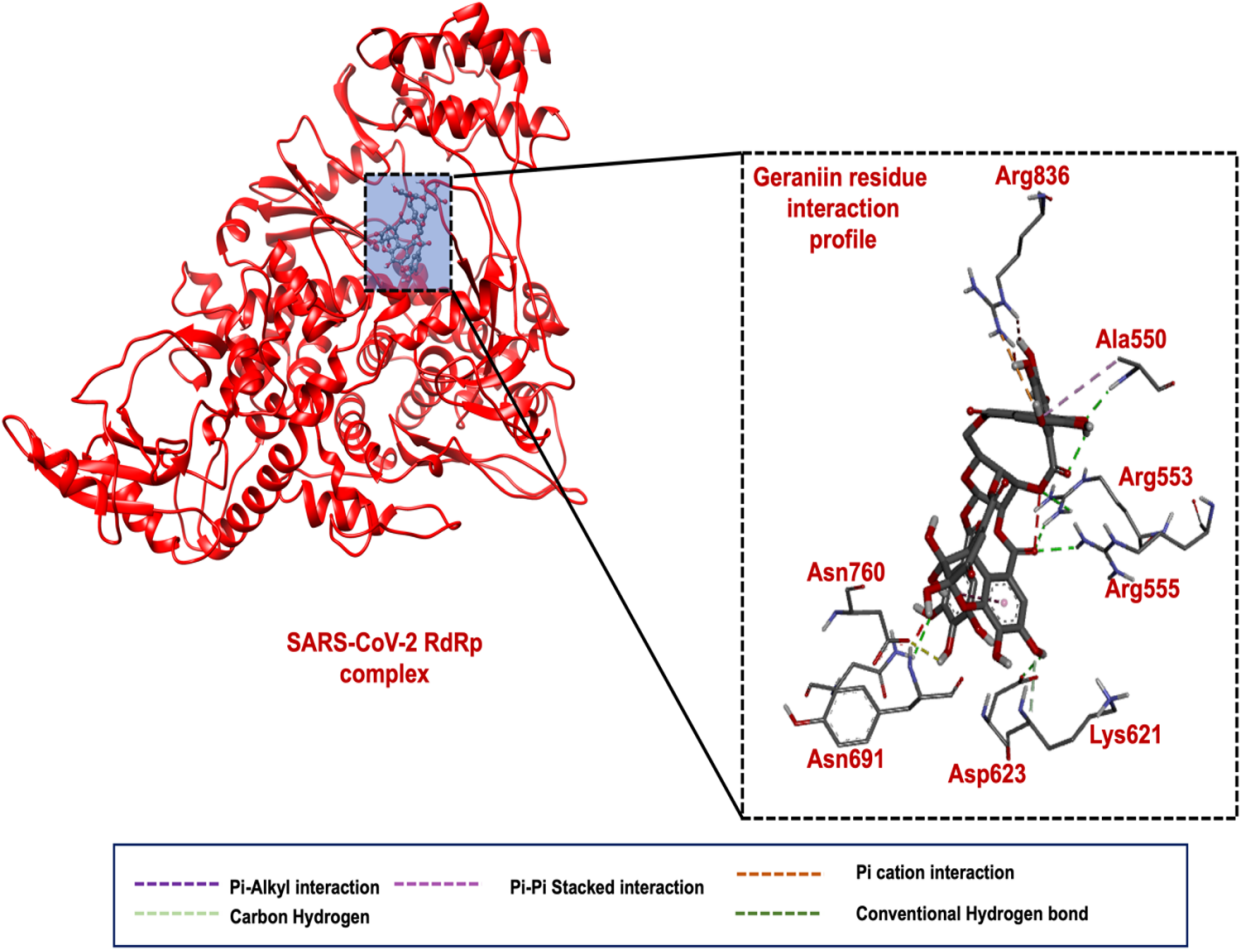
(**A**) 3D representation of RdRp bound with Geraniin. (**B**) 3D ligand interaction plot of the RdRp bound with Geraniin.

As shown in Fig. 2, Geraniin, which exhibited the most favourable docking score amongst the studied phytochemicals against SARS-CoV-2 RdRp, forms a pi-alkyl bond with Arg550, a conventional hydrogen bond with both Arg555 and Ala553, and a pi-cation interaction with Arg836. Geraniin also forms a conventional hydrogen bond with Asn691, Asn760, and Asp623 and a carbon-hydrogen bond interaction with Lys621 in deeper regions of the inhibitor binding site. These interactions collectively accounted for the favourable docking score of − 10.4 kcal/mol as estimated since these interactions anchored Geraniin within the binding pocket after assuming a favourable binding conformation. A comparison of the docking score of Geraniin (− 10.4 kcal/mol) with that of the docking score of Remdesivir (− 8.2 kcal/mol), a reported SARS-CoV-2 RdRp inhibitor^44^, within the inhibitor binding site of SARS-CoV-2 RdRp, revealed that Geraniin showed a relatively higher negative docking score for Geraniin than Remdemsivir. The suggested a relatively stronger binding potential of Geraniin towards SARS-CoV-2 RdRp than Remdesivir and thereby warranted further investigations of the compound Geraniin anti- SARS-CoV-2 potential.

#### SARS-CoV-2 receptor binding domain-Geraniin complex binding mechanism

**Figure 3:**
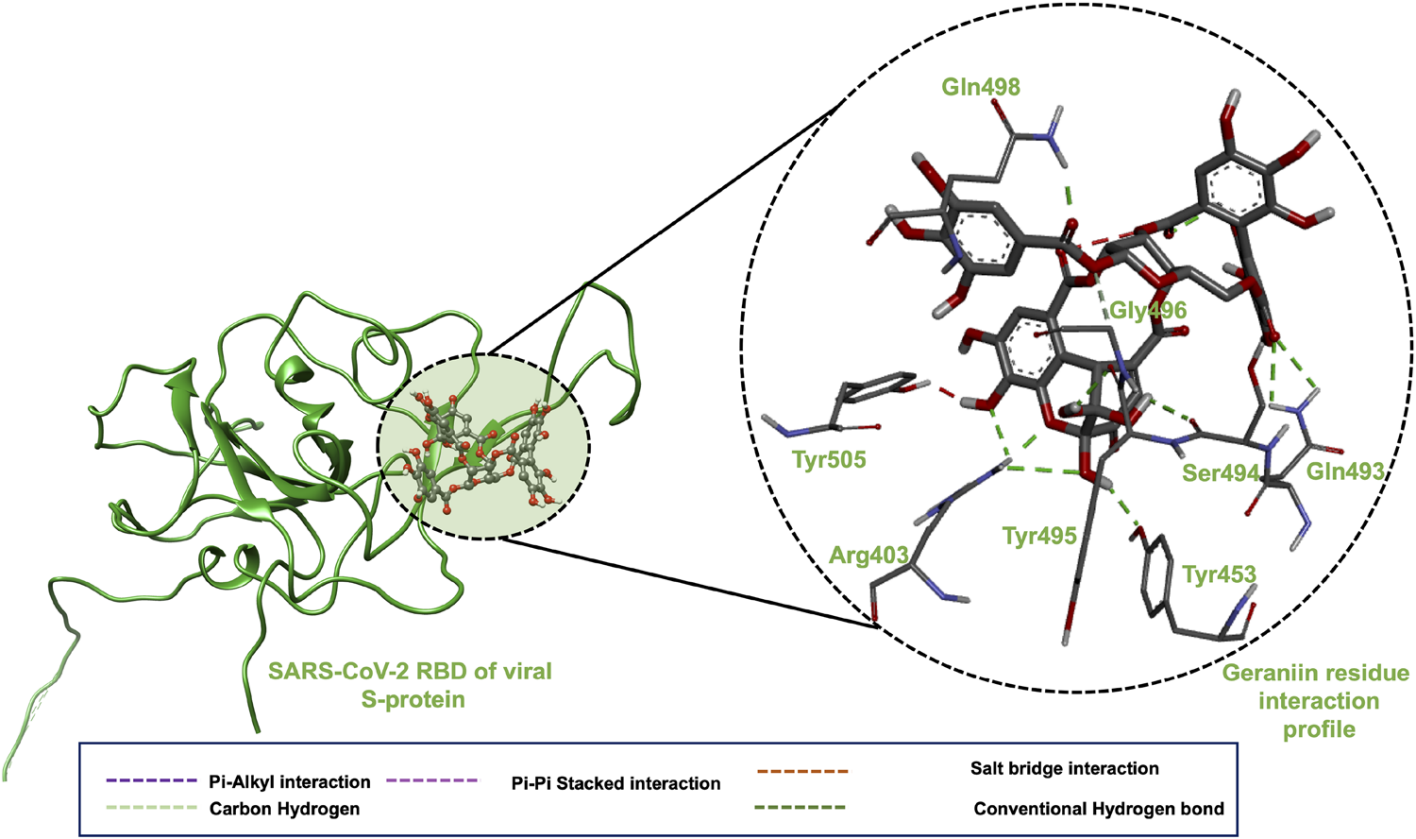
(**A**) 3D complex of RBD of viral S-protein and Geraniin. (**B**) 3D ligand interaction plot of the RBD of viral S-protein complexed with Geraniin.

As shown in Table 4, Geraniin also exhibited the highest docking score toward the RBD of SARS-CoV-2 viral S-protein. By examining its residue interaction profile with the RBD, we explored its possible binding mechanism. A successful blockage of the RBD of viral S-protein by Geranin could impede the binding of RBD of viral S-protein and SARS-CoV-2. As shown in Fig. 3, Geraniin is engaged in a vast network of interactions, notably, conventional hydrogen bond interactions were formed with Arg403, Tyr495, Tyr453, Ser494, Gln493, Gln498 and Tyr505, while a carbon-hydrogen interaction is observed with Gln498. These strong conventional hydrogen interactions could anchor Geraniin within the binding pocket to ensure its stability for favourable binding and significant interruption of the activity of RBD of the viral S-protein. The interacting residues were also consistent with dominant residues reported by several studies and residues crucial to the inhibition of the RBD of viral S-protein^137^. These structural inhibitory potentials provided in addition to the previously reported anti-viral activity of Geraniin^90,92^ necessitates a further investigation of Geraniin as a possible inhibitory candidate of the receptor-binding domain of viral S-protein.

#### Identified hits exhibit favorable binding free energy towards SARS-CoV-2 3CL^pro^, RdRp and RBD of viral S protein

Inhibitor stability within the binding pocket is very crucial in determining biological processes with significant pharmaceutical implications. Therefore, to establish the stability of the identified hits within the respective SARS-CoV-2 target, we assessed their binding free energy over the simulation period using the MMPB-SA approach since binding affinities from molecular docking are inconclusive. The MM/PBSA calculations also allowed for a quantitative determination of absolute binding affinities of the identified hits^138^. The calculated binding free energies allowed for a thorough understanding of the mechanism by which the respective SARS-CoV-2 targets recognize the identified hits^139^. As shown in Table 3, the estimated binding free energies of Geraniin towards SARS-CoV-2 RdRp and RBD of viral S protein were − 25.87 kcal/mol and − 21.74 kcal/mol, respectively, while the binding free energy of 2-O-Caffeoyl-(+)-allohydroxycitric acid against 3CL^pro^ was − 32.00 kcal/mol. Overall, all three compounds bound exhibited strong binding affinity towards their respective target, corroborating with the strong interaction bonds elicited binding pockets as revealed in the interaction dynamics. 2-O-Caffeoyl-(+)-allohydroxycitric acid exhibited almost similar binding free energy with Ritonavir, a reported 3CL^pro^ inhibitor^140^, which showed a total binding free energy of − 32.34 kcal/mol. Also, a comparison of the binding free energy of Geraniin to the known SARS-CoV-2 RdRp inhibitor, Remdesivir, showed that Geraniin exhibited a relatively lower binding free energy than Remdesivir, which demonstrated binding free energy of − 33.34 kcal/mol. This generally favourable binding affinity of the studied phytochemicals in addition to structural insights provided prompts a need for further investigation as potential inhibitors.

**Table 3:** MM/PBSA-based binding free energy profile of identified hit compounds against respective SARS-CoV-2 therapeutic targets.

Complexes	Energy components (kcal/mol)				
	${\Delta E}_{{{\text{vdw}}}}$	${\Delta E}_{{{\text{ele}}}}$	${\Delta G}_{{{\text{gas}}}}$	${\Delta G}_{{{\text{sol}}}}$	${\Delta G}_{{{\text{bind}}}}$
*2OCA-3CL*^*pro*^	− 32.06 ± 0.16	− 55.91 ± 0.58	− 87.97 ± 0.67	55.12 ± 0.39	− 32.00 ± 0.31
*Ritonavir-3CL*^*Pro*^	− 49.71 ± 0.33	− 40.35 ± 0.65	− 9.35 ± 0.84	− 22.99 ± 0.59	− 32.34 ± 0.34
*Geraniin-RBD*	− 36.22 ± 0.23	− 23.12 ± 0.49	− 59.34 ± 0.66	37.60 ± 0.42	− 21.74 ± 0.27
*Geraniin-RdRp*	− 24.00 ± 0.84	− 49.28 ± 1.74	− 73.29 ± 2.57	47.41 ± 1.67	− 25.87 ± 0.91
*Remdemsivir-RdRp*	− 44.4 ± 0.3	− 38.7 ± 0.8	− 83.2 ± 1.0	49.5 ± 0.7	− 33.4 ± 0.4

ΔE_ele_ = electrostatic energy; ΔE_vdW_ = van der Waals energy; ΔG_bind_ = total binding free energy; ΔG_sol_ = solvation free energy; ΔG_gas_ = gas phase free energy.

### Assessing the structural and conformational changes of SARS-CoV-2 therapeutic targets upon binding of Geraniin and 2-O-Caffeoyl-(+)-allohydroxycitric acid

As a reliable and widely employed computational technique, molecular dynamics simulations were used to conduct a time-dependent prediction of the structural and conformational motions that occur on the SARS-CoV-2 therapeutic targets upon the binding of the identified bioactive compounds^141–143^. Any observed structural changes on these SARS-CoV-2 targets could contribute to the potential inhibitory activity of the compounds. With an adequate 200 ns MD simulation period, we calculated the root mean square deviation (RMSD)^144^ and root mean square fluctuation (RMSF)^143,145^ to assess conformational stability and residue flexibility of each of the therapeutic targets as associated with the inhibitor binding of the phytochemicals.

#### 2-O-Caffeoyl-(+)-allohydroxycitric acid-binding perturbs 3CL^pro^

**Figure 4:**
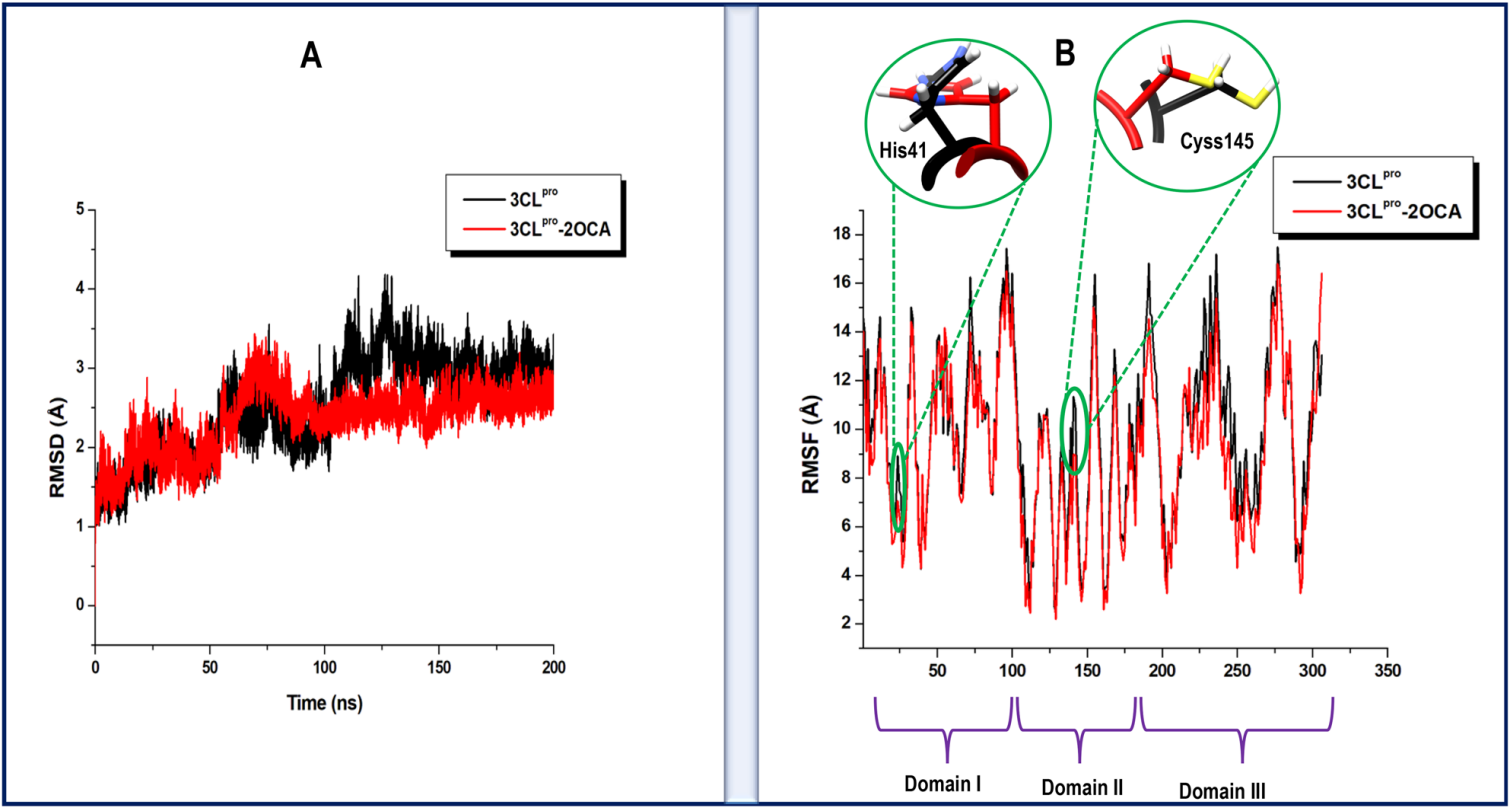
(**A**) Comparative root mean square deviation(RMSD) plots of the 2-O-Caffeoyl-(+)-allohydroxycitric acid bound 3CL^pro^ (red) and unbound (black), showing that inhibitor stabilized the 3CL^pro^. (**B**) Comparative root mean square fluctuation (RMSF) plots of the 2-O-Caffeoyl-(+)-allohydroxycitric acid bound 3CL^pro^ (red) and unbound (black). Insert highlights 3D representation of the variation in flexibility of the catalytic dyad of 3CL^pro^ in both bound and unbound conformations.

Several recent reports have investigated the conformational dynamics of unliganded of SARS-CoV-2 3CL^pro^, including a recent molecular dynamics simulations study by Suarez and Diaz (2020)^146^ where they revealed that the domain III of 3CL^pro^ is generally unstable while the presence of peptide substrate, induces a stable interdomain arrangement in the monomeric conformation of the protease. These conformational changes are the hallmarks of the SARS-CoV-2 target dynamics and correlate with its overall functioning^146^. By calculating the RMSD of the C-α atoms of 3CL^pro^ over the 200 ns simulation period, the impact of the binding of 2-O-Caffeoyl-(+)-allohydroxycitric acid on the stability of 3CL^pro^ was assessed. The stability of the protein structure is crucial in the maintenance of its function^147^. As shown in Fig. 4A and B, both unbound and bound simulated models of 3CL^pro^ converged around 75 ns after an initial jump in deviation due to the expansion of atoms. On average, the unbound conformation of 3CL^pro^ exhibited an RMSD of 2.53 Å while the inhibitor bound conformation exhibited RMSD of 2.43 Å. This suggested that the binding mechanism of 2-O-Caffeoyl-(+)-allohydroxycitric acid involved a subsequent increase in the stability of 3CL^pro^. The stabilized conformation of 3CL^pro^ upon the binding of 2-O-Caffeoyl-(+)-allohydroxycitric acid could further facilitate a favorable interaction with crucial active site residues to impede the functions of 3CL^pro^. Root Mean Square Fluctuation (RMSF), which provided molecular insights on the flexibility of each of the 306 amino acid residues of 3CL^pro^, was estimated as presented in Fig. 4B. The unbound simulated model of 3CL^pro^ exhibited an average RMSF of 9.90 Å, while the 2-O-Caffeoyl-(+)-allohydroxycitric acid bound model showed a relatively lower average RMSF of 9.16 Å. The result suggested that the binding of 2-O-Caffeoyl-(+)-allohydroxycitric acid impeded the flexibility of individual amino acids of 3CL^pro^, consistent with the relatively lower average RMSD of the bound conformation as observed. This restricted residue flexibility could interfere with the essential residue mobility required for the function of 3CL^pro^. From the RMSF and RMSD calculations, it could be concluded that the binding of 2-O-Caffeoyl-(+)-allohydroxycitric acid is characterised by a stabilized structural conformation and an impeded residue flexibility which in turn interfere with essential 3CL^pro^ mobility.

#### Geraniin binding distorts conformational integrity of SARS-CoV-2 RdRP

**Figure 5:**
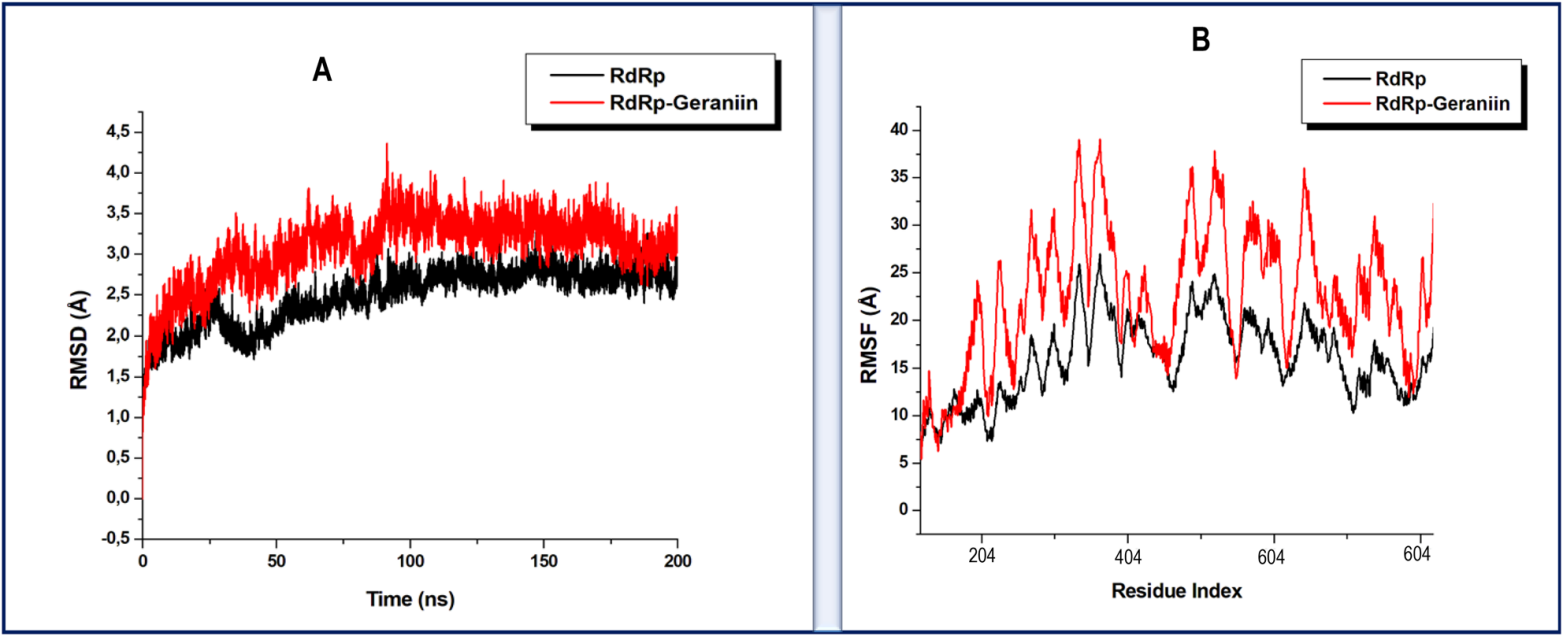
(**A**) Comparative root mean square deviation plots of the Geraniin bound RdRp (red) and unbound (black), showing that Geraniin induced an unstable conformation in RdRp. (**B**) Comparative root mean square fluctuation plots of the Geraniin bound RdRp (red) and unbound (black) showing increased residue flexibility upon Geraniin binding.

A recent comparative molecular dynamics simulations study by Koulgi et al. (2020)^148^ where the unbound and Remdesivir-complexed structures of SARS-CoV-2 RdRp showed the blocking of the template entry site upon Remdesivir binding^148^. Their report further revealed that Remdesivir binding is characterised by structural instability and increased residue flexibility. To ascertain the inhibitory potential of Geraniin against RdRp, we also assessed the conformational dynamics of RdRp upon Geraniin binding. In a similar mechanism as Remdesivir, the binding of Geraniin also increased the deviation of c-alpha atoms of RdRp implying its structural instability as shown in Fig. 5A, whereby a relatively higher average RMSD of 3.08 Å was calculated for the Geraniin bound RdRp. The unbound RdRp, on the other hand, exhibited an average RMSD of 2.5 Å. Likewise, as shown in Fig. 5B, the binding of Geraniin also induced prominent residue fluctuations, as was reported for Remdesivir binding in the study by Koulgi et al*.* (2020)^148^*.* An average RMSF of 32.01 Å was estimated for the Geraniin bound RdRp, while an average RMSF of 21.70 Å was calculated for the unbound conformation. In summary, it could be inferred that the binding of Geraniin induced structural changes on SARS-CoV-2 RdRp in a similar mechanism to Remdesivir. As such, Geraniin could further be investigated as a potential inhibitor of SARS-CoV-2 RdRp.

#### Geraniin binding influences the receptor accessibility or inaccessibility of the spike protein

**Figure 6:**
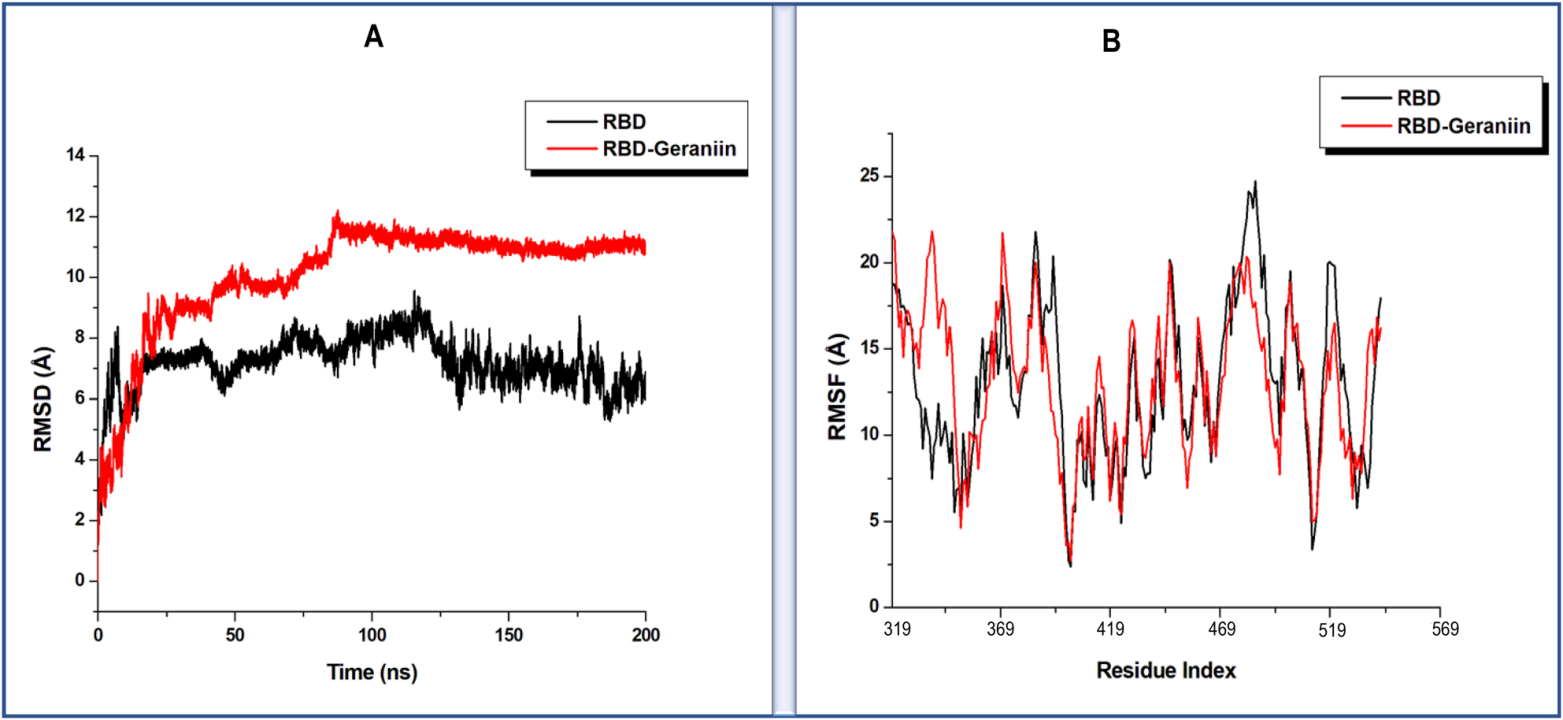
(**A**) Comparative root mean square deviation plots of the Geraniin bound to RDB (red) and unbound (black), showing that Geraniin induced an unstable conformation in RBD. (**B**) Comparative root mean square fluctuation plots of the Geraniin bound to RDB (red) and unbound (black), showing a slightly increased residue flexibility upon Geraniin binding.

According to a recent report by Gur et al. (2019)^149^, the down and up positions of SARS-CoV-2 RBD can interfere with the accessibility of the spike protein by controlling its open (receptor accessible) and closed (receptor inaccessible) positions. Therefore, it is evident that any conformational changes of RBD induced by a bound inhibitor could influence any intended therapeutic inhibition. A calculation of the RMSD of the simulated RBD models as presented in Fig. 6A and B revealed that the unbound conformation of RBD showcased an average RMSD of 7.20 Å. At the same time, the Geraniin bound RBD showed an average RMSD of 10.17 Å. The significantly higher average RMSD of the bound conformation may suggest that the binding of Geraniin possibly increased the deviation of c-α atoms and hence subsequently decreased the conformational stability of RBD. The flexibility of the individual amino acids of RBD was assessed. As shown in Fig. 6, an average RMSF of 12.96 Å and 13.08 Å were calculated for the unbound and inhibitor-bound conformation of RBD, respectively. Although the difference in average residue fluctuations between the bound and unbound conformations was minimal, the relatively higher average RMSF in the Geraniin bound structure confers with increased residue flexibility, suggesting that the binding of Geraniin distorted the residue integrity of RBD, which subsequently increased the residue motions as observed. This increased residue mobility of RBD upon Geraniin binding could in turn favour a down and up motion of RBD and hence possibly influence the receptor accessibility or inaccessibility of the spike protein as postulated by Gur et al. (2019).

### Assessing the pharmacokinetic properties of Geraniin and 2-O-Caffeoyl-(+)-allohydroxycitric acid

The physicochemical and pharmacokinetic features of drugs are very crucial to their overall therapeutic success. As such, we analysed the physicochemical and pharmacokinetic properties of Geraniin and 2-O-Caffeoyl-(+)-allohydroxycitric acid using the online platform SwissADME^120^. An in silico assessment of these properties, notably absorption, distribution, metabolism, and excretion, offers insights into the pharmacokinetics of a given small molecular inhibitor in vivo while minimizing the risk of being disapproved during the late stages of the drug development^118,119^. In Table 4, we presented the physicochemical and pharmacokinetic properties of Geraniin and 2-O-Caffeoyl-(+)-allohydroxycitric acid as assessed from SwissADME. An initial assessment of the drug-likeness of Geraniin and 2-O-Caffeoyl-(+)-allohydroxycitric acid by the Lipinski's rules of five^150^ (molecular weight (MW) ≤ 500 g/mol], Log P ≤ 5, H-bond donors (HBD) ≤ 5 and H-bond acceptors (HBA) ≤ 10) revealed that Geraniin violated three rules whereas 2-O-Caffeoyl-(+)-allohydroxycitric acid violated two rules. This indicated that based on Lipinski's rules of 5, both compounds were not drug-like. However, this was not surprising since, generally, natural products have been shown to not adhere to Lipinski's rule of 5^150^. Interestingly, however, Geraniin possessed a log P o/w of − 1.71, whereas 2-O-Caffeoyl-(+)-allohydroxycitric acid showed a log P o/w of − 0.65 and a molecular weight of 370.27 g/mol as showed in Table 4. Prediction of the compounds’ lipophilicity by assessing their LogP o/w showed that both compounds showed poor lipophilicity. The lipophilicity of a compound significantly influences pharmacokinetic properties such as the absorption, distribution, permeability, and routes of drug clearance. A favourable log P usually ranges between 3 and 5. A low LogP of a compound often indicates a lower membrane permeability and poor absorption^150–152^. With a logP o/w of − 1.71 and − 0.65, it suggests that both compounds are unable to permeate lipid membranes and will exhibit poor bioavailability. Nonetheless, with a large MW of 952.65 kcal/mol, its synthetic fragmentation into smaller simpler compounds could increase its bioactivity and decrease toxicity^153^. Our results indicate that, Geraniin and 2-O-Caffeoyl-(+)-allohydroxycitric acid exhibited a poor pharmacokinetic properties. However, further experimental explorations based on the favourable binding mechanism could lead to the discovery of novel SARS-CoV-2 inhibitors.

**Table 4:** Physicochemical and pharmacokinetic properties of Geraniin and 2-O-Caffeoyl-(+)-allohydroxycitric acid.

	Geraniin	2-O-Caffeoyl-(+)-allohydroxycitric acid
Molecular weight (g mol^−1^)	952.64 g/mol	370.27 g/mol
Molecular formula	C_41_H_28_O_27_	C_15_H_14_O_11_
Lipophilicity (logP)	− 1.71	− 0.65
Water solubility	Soluble	Soluble
Human gastrointestinal tract (GIT) absorption	Low	Low
Blood–brain barrier (BBB) permeability	No	No
Bioavailability score	0.17	0.11
Hydrogen bond (donors/acceptors)	14/27	6/11
Drug likeness (Lipinski)	No	No
Leadlikeness	No	No
BOILED-egg representation of lipophilicity and polarity		

## Conclusion

In conclusion, this in silico study identified two phytochemical compounds, Geraniin and 2-O-Caffeoyl-(+)-allohydroxycitric acid as potential inhibitor candidates against SARS-CoV-2. Using Molecular Mechanics/Poisson-Boltzmann Surface Area (MM/PBSA) approach to calculate binding free energy, Geraniin exhibited binding energy (ΔG_bind_) of − 25.87 kcal/mol and − 21.74 kcal/mol towards SARS-CoV-2 RdRp and RBD of viral S protein, respectively. 2-O-Caffeoyl-(+)-allohydroxycitric acid, on the other hand, exhibited a ΔGbind of − 32 kcal/mol towards 3CL^pro^. The binding of both Geraniin and 2-O-Caffeoyl-(+)-allohydroxycitric acid were characterised by strong interactions with respective SARS-CoV-2 therapeutic targets, suggesting an inhibitory potential against SARS-CoV-2 RNA-dependent polymerase, 3CL^pro^ and RBD of the viral S-protein. Molecular Dynamics (MD) simulations further revealed crucial structural changes induced by Geraniin and 2-O-Caffeoyl-(+)-allohydroxycitric acid, which possibly interfered with the functions of the SARS-CoV-2 proteins. Notable structural changes included increased residue flexibility and distortion of the structural integrity of SARS-CoV-2 RdRp RBD upon Geraniin binding. The binding of 2-O-Caffeoyl-(+)-allohydroxycitric acid was, however, shown to stabilize and impede the residue flexibility of 3CL^pro^. The molecular insights provided present Geraniin and 2-O-Caffeoyl-(+)-allohydroxycitric acid and potential SARS-CoV-2 inhibitor candidates. However, an extensive experimental evaluation is recommended to further establish their SARS-CoV-2 inhibitory potential.

## Study limitation

Authors acknowledge that computational molecular docking analysis and MD simulations have their limitations, and that further laboratory and clinical studies are needed to validate the inhibitory effects of these candidates against SARS-CoV-2 as potential drugs for COVID-19.

## Future perspective/implications of results

To the best of our knowledge, this is the first account of in silico study aimed at phytochemical compounds; Geraniin and 2-O-Caffeoyl-(+)-allohydroxycitric acid isolated from ethanolic leaf extract of *S. mombin*, against SARS-CoV-2 RNA-dependent polymerase, 3CL^pro^, and receptor binding domain of viral S-protein.

It is therefore envisaged that interest will be generated for in vitro study of the inhibitory potency of the crude ethanolic extract of *S. mombin* and/or pure compounds of Geraniin and 2-O-Caffeoyl-(+)-allohydroxycitric acid towards the discovery of novel SARS-CoV-2 therapeutics.
